# Simple and multiplexed tracking of bacterial growth in double emulsion droplets

**DOI:** 10.1371/journal.pone.0362111

**Published:** 2026-11-04

**Authors:** Sebastián Somolinos Cedeño, Samuel Thompson, Polly Fordyce, Drew Endy

**Affiliations:** 1 Department of Microbiology and Immunology, Stanford University, Stanford, California, United States of America; 2 Department of Bioengineering, Stanford University, Stanford, California, United States of America; 3 Department of Genetics, Stanford University, Stanford, California, United States of America; 4 Sarafan ChEM-H Institute, Stanford University, Stanford, California, United States of America; 5 Chan Zuckerberg Biohub, San Francisco, California, United States of America; 6 Center for International Security and Cooperation, Freeman Spogli Institute, Stanford, California, United States of America; 7 Hoover Institution, Stanford, California, United States of America; Kyushu University Faculty of Agriculture Graduate School of Bioresource and Bioenvironmental Sciences: Kyushu Daigaku Nogakubu Daigakuin Seibutsu Shigen Kankyo Kagakufu, JAPAN

## Abstract

Droplet-based microfluidics enable researchers to observe phenotypic heterogeneity within complex biological mixtures through parallel encapsulation of individual samples followed by imaging. Observing or quantifying dynamic heterogeneity remains challenging due to complexities associated with trapping and tracking many individual droplets. Current approaches for time-lapse imaging require specialized devices with droplet traps that limit accessibility and throughput. Here, using readily available materials and software, we demonstrate a simple method for stabilizing and monitoring many, individual droplets for up to 12 hours. We leveraged our method to track bacterial growth within droplets in a high-throughput manner for over 6 hours. Our method allows tracking the changes and variation in growth rate within and across droplets, revealing heterogeneity in growth patterns hidden in batch assays. Improving the affordability and throughput of time-dependent phenotyping assays helps to advance biological discovery and biotechnology innovation.

## Introduction

Droplet microfluidic technologies enable high-throughput imaging of individuals comprising complex populations [1–5]. Via highly parallelized compartmentalization, individuals suspended in a continuous aqueous phase can be isolated and encapsulated within an external oil shell with high precision and speed [6], creating many distinct and independent samples.

Double-Emulsion (DE) droplets have gained attention relative to single-emulsion (SE) approaches [7–9]. DE droplets are composed of an inner aqueous phase, an oil shell, and an outer aqueous phase (Fig 1A). Surfactants stabilize each water-to-oil interface, lowering the surface tension to allow the droplets to self-assemble in a microfluidic generator and providing sufficient repulsion to protect droplets from coalescing. The outer aqueous phase further reduces the likelihood of droplet merging and allows for nutrient exchange. DEs also enable droplet isolation through Fluorescence Activated Cell Sorting (FACS), circumventing the complex set-ups required for sorting single-emulsion droplets through Fluorescently Activated Droplet Sorting (FADS) [10].

**Fig 1:**
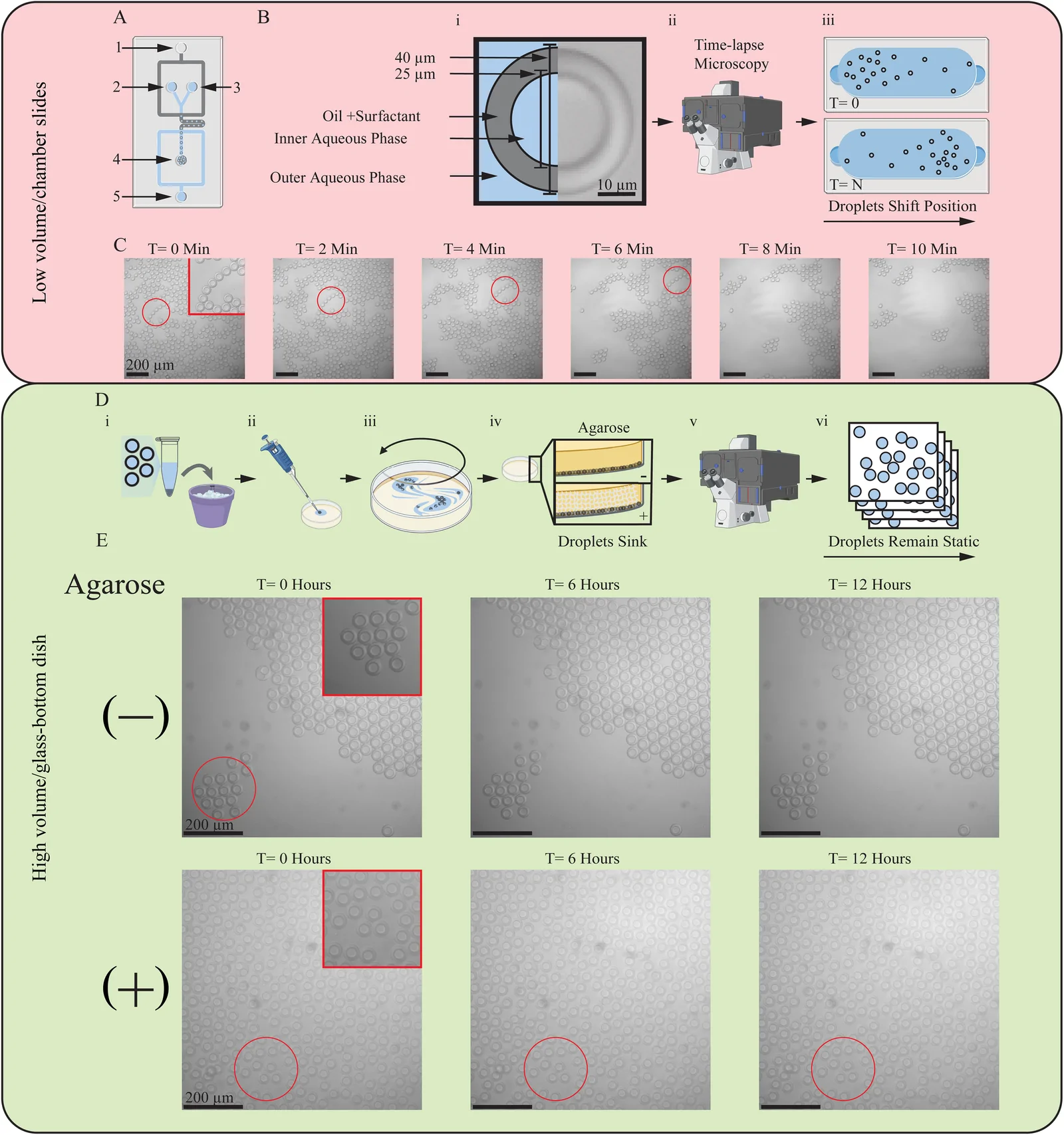
A simple method enabling simple long-term tracking of double emulsion (DE) droplets. (A) Schematic of DE-droplet generator device. (B) Schematic showing the problem addressed in this work: 1) DE-droplets (layers and dimensions included) are generated, 2) introduced into a temperature-controlled microscope, and 3) lost during imaging due to positional shifting within the chamber slide. (C) Bright field microscopy images of a single field of view (FOV) containing DE-droplets over time; Red circle indicates the position of a particular set of droplets as they migrate. (D) Schematic of experimental pipeline for immobilizing DE droplets in agarose followed by time-lapse imaging: 1) generate droplets and keep on ice until imaging, 2) inject droplets at the bottom of a glass microscopy dish filled with molten agarose, 3) gently swirl the dish, 4) allow agarose to solidify for 5 minutes, 5) place dish on the microscope stage, and 6) perform time-lapse imaging of desired FOVs. (E) Bright field microscopy images showing droplets immobilized using different agarose concentrations at 0 hours (left), 6 hours (middle), and 12 hours (right).

Researchers have realized a wide variety of enzymatic and cellular screens by sorting microfluidic droplets to isolate and recover individual samples expressing desired properties [11–18]. DE droplets specifically have been used to isolate and enrich diverse biological entities including DNA molecules, enzymes, viruses, and prokaryotic and eukaryotic cells [19–23]. Of note, DE droplets have been used to partition populations across individual compartments, enabling growth in the absence of competition and thus facilitating measurement of low-fitness or uncommon variants that would otherwise be masked by faster-growing subpopulations if tested in bulk [24].

DE-droplets can also be useful during imaging. Microscopy of droplets can provide quantitative information about time-dependent processes taking place within thousands of individual droplets in parallel [25]. Heterogeneous behaviors that are obscured when averaging across droplets or limiting data collection to static imaging can also be revealed [26]. Parallel microencapsulation of individuals enables access to the diversity of phenotypes underlying population-level measurements, revealing insights into the dynamic range of certain kinetic processes. Continuous monitoring of specific droplets could also help screen enzyme variants for turnover speed or help determine the prevalence of a sub-population of growth-deficient cells. Beyond real-time observations of emergent dynamic heterogeneity, time-lapse DE droplet imaging could provide information critical in the process of developing, troubleshooting, and improving novel high-throughput screens [27,28]. Taken together, simple and scalable pipelines for tracking DE droplets could expand the accessibility and usefulness of high-throughput imaging for complex biological samples [29].

Immobilization of droplets is well established by several mature methods [29], but each requires microfluidic device fabrication. We show that comparable stability is achievable with a device-free method using only off-the-shelf materials, in a manner compatible with maximized droplet packing density. A common challenge in droplet tracking is that over time droplets tend to move, shifting in and out of the field of view and focal plane. Because droplets move in response to external forces (e.g., evaporation, jostling) existing approaches often rely on repeated loading of sample aliquots at different time points. While static imaging can capture population-level measurements, doing so precludes longitudinal tracking of individual droplets, which is essential in understanding the underlying causes of population dynamics and heterogeneity [26]. Collecting droplet tracking data should ideally be simple, with the throughput only limited by the size and number of captured fields of view (FOV).

Previous studies performing time-lapse imaging of droplets have required specialized secondary devices containing droplet traps [2,5,30–35]. Designing and operating devices with droplet traps is challenging, often requiring fabrication of new multi-height devices with tailored trap geometries [26] and either continuous flow [36] or valves [37] for operation. The manufacturing process for such devices requires expertise, access to a clean room, and often several rounds of iteration during fabrication. Additionally, most computational droplet tracking methods rely on custom software, complicating accessibility and reproducibility. Custom tracking algorithms are often tailored to a particular use case and can be computationally expensive, taking up to four days to process an experimental run [31]. Other methods using broadly accessible software rely on secondary trapping devices and have yet to be applied to tracking of DE droplets [2].

We developed a simplified imaging and tracking pipeline to streamline multiplexed monitoring of DE droplets. We only used readily available consumables and software to help reduce barriers to future adoption. As a benchmark, we monitored the increase in bacterial cell density within droplets over time, expecting an initial lag to be followed by an exponential increase, and ultimately becoming stationary. Plotting the resulting data as a line chart revealed a sigmoidal curve typical of bulk-based assays. Beyond recapitulating expected cell behaviors, our method enabled tracking of bacterial growth within hundreds of DE droplets in a single FOV over several hours, and processing of the resulting data within minutes. Furthermore, we demonstrated compatibility of DE droplets with agarose trapping during high-throughput imaging and tracking.

## Results

### Use of a glass-bottom dish, increased sample volumes, and agarose help minimize droplet drift

To demonstrate the severity of the droplet shifting issue in trap-less imaging set-ups, we tested how long we could track and image DE droplets without immobilization. Droplets generated using a standard PDMS microfluidic chip (Fig 1A) were roughly 40 µm in diameter and contained a ~ 25 µm inner core of PBS surrounded by a shell of HFE7500 oil with 5% PicoSurf surfactant (Fig 1B, i). We generated a monolayer of DE droplets suspended in PBS by depositing them on a Countess chamber slide (Thermo Fisher Scientific), and then imaged droplets via time-lapse, bright-field microscopy at two-minute intervals (Fig 1B, ii). Images of droplets showed substantial displacement over time, with droplets from a packed field of view gradually moving out of the FOV (Fig 1B, iii).

The speed and magnitude of displacement was severe enough to preclude computationally tracking the identity of any given droplet, even at high temporal resolution. Most droplets present in the first frame (t = 0) drifted out of the original FOV by the fifth frame (t = 10 minutes) (Fig 1C).

We pursued two solutions to limit droplet movement. First, we attempted an optimized “trap-less” method that kept droplets unconfined. To prototype a simple implementation of a trap-less imaging set-up, we used a glass bottom dish (D35C4-20–0-N, Cellvis) and increased the relative volume of the outer buffer (Fig 1D). We reasoned that using glass would help reduce shifting due to temperature-induced surface bending and increased buffer volume would reduce the impact of evaporation. The high density of DE-droplets ensured they would sink and form a monolayer at the glass interface, reducing vertical movement while maximizing imaging throughput. Second, we tested the compatibility of DE droplets with agarose-based trapping (Fig 1D and S1 Fig). We hypothesized that droplets embedded in agarose would remain fixed in a FOV for the duration of a growth experiment without requiring additional device fabrication. We added agarose at 0.25 to 1% w/v into our outer buffer (PBS). Through microscopy, we observed consistent stability in droplet integrity, focal plane, and relative position within the FOV for up to 12 hours, regardless of agarose presence (Fig 1E and S2 Fig.).

Our results suggest that DE droplets are compatible with agarose trapping and that either pipeline was robust enough to prevent excessive droplet shifting for up to 12 hours. Taken together, our findings suggested downstream compatibility with multiplexed imaging and tracking of time-dependent processes in DE droplets, such as bacterial growth.

### Fluorescence imaging of DE-droplets reveals differences in cell density

Next, we were curious about the sensitivity of our simplified, single-plane imaging set-up and whether it could detect differences in cell densities well enough to detect growth. At low cell densities, individual bacteria could be difficult to detect inside droplets; at high cell densities, it could be challenging to quantify changes in the absolute number of bacteria. As a proof of concept, we experimentally mocked-up expected conditions at the start and end of a bacterial growth experiment by encapsulating cultures at medium (OD = 0.84) and high (OD = 9.4) density (working OD’s 0.42 and 4.7, respectively, after 1:1 on-chip dilution with sterile LB) and acquiring a single static image (Fig 2 and S3 Fig.). We selected these cell densities because an OD of 0.84 would lead to a distribution of cells ranging from 0 to 4 cells per droplet (CPD), a range chosen to be easily countable and amenable to the detection of single cells. We chose an OD of 9.4 for a high level of cells (10–40 per droplet) because this density would result from three to four cell doublings yet remain well below the theoretical maximum packing of cells per droplet (less than 8000 cells per droplet).

**Fig 2:**
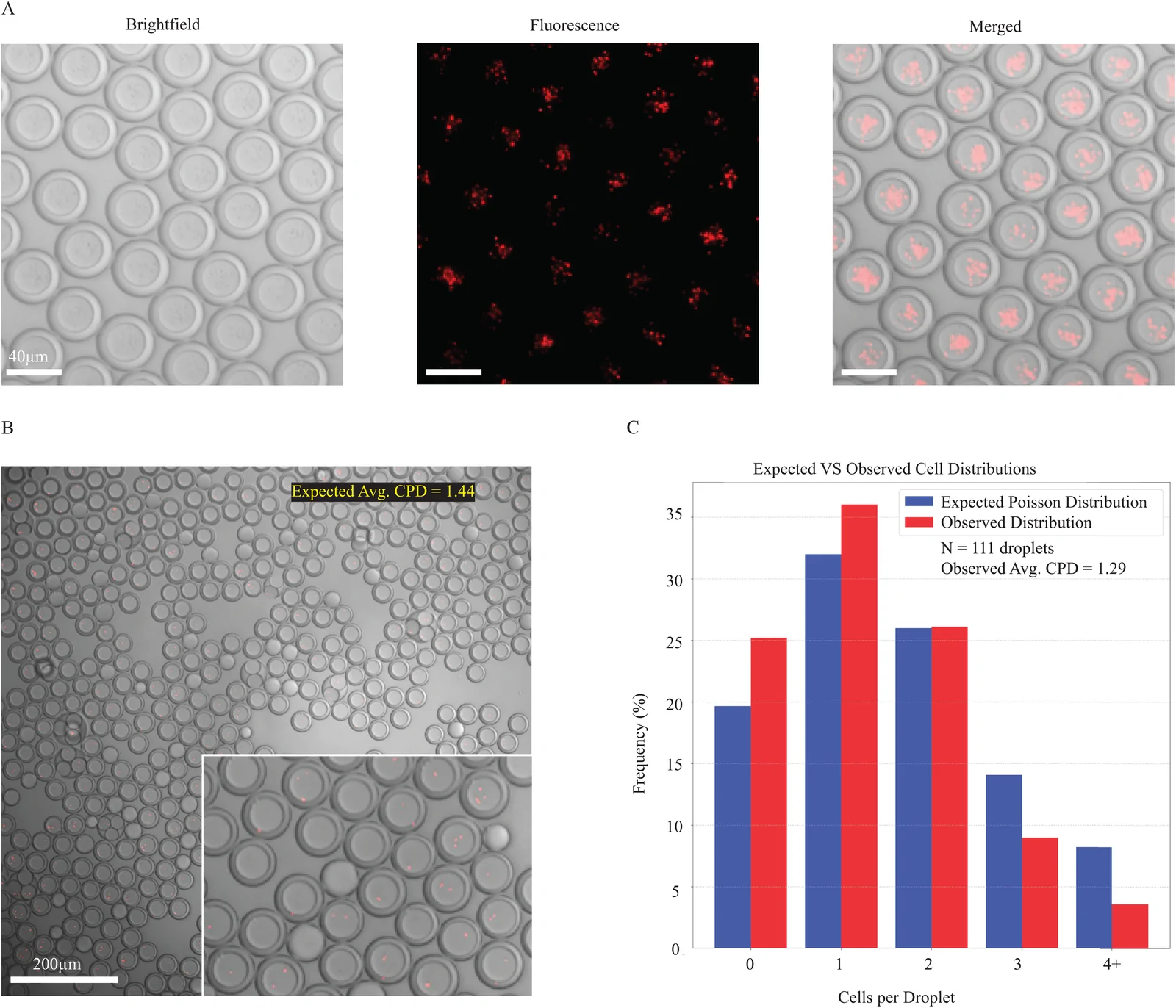
Fluorescence imaging of DE-droplets can reveal differences in the number of cells per droplet. (A) Representative bright field (left), fluorescence (middle), and merged (right) images of a single DE droplet containing mScarlet1-expressing *E. coli* cells loaded under the medium density regime. (B) Example merged image showing a single field of view and zoomed in view (inset) of DE droplets containing mScarlet1-expressing *E. coli* cells loaded under the medium density regime. (C) Histograms showing expected (blue) and observed (red) distributions of the number of cells per droplet.

We encapsulated bacteria in DE droplets by introducing LB-grown cultures and LB alone into the first and second aqueous inner inlets of the microfluidic chip, respectively. The encapsulated *E. coli* constitutively expressed a fluorescent marker (mScarlet1), allowing us to observe cell presence and relative abundance by quantifying pixel intensities (Fig 2A). Discerning single cells in the high-density regime proved challenging, yet the presence of distinct puncta within medium-density droplets suggested we could resolve individual cells (Fig 2B).

To further explore the possibility of single-cell detection, we manually counted cells within 111 medium-density droplets and compared our findings to the expected distribution of CPD assuming stochastic loading (Fig 2C). Counts were binned as 0, 1, 2, 3 and 4 + , since individual puncta within a droplet become hard to count above roughly four cells as pixel puncta begin to overlap. The averages (λ) of both the observed and expected Poisson distributions within medium density droplets agreed with one another (expected λ = 1.44, observed λ = 1.29 ± 0.11 (SE), 95% CI [1.08, 1.50]), with the interval containing the value predicted from input optical density (z = −1.39, p = 0.16, two-sided; z expresses the difference between observed and predicted λ in standard errors of the observed λ, and p is the probability of a departure at least this large if the true mean equaled the predicted value). The modest shortfall relative to the predicted λ is consistent with the ~ 90% per-cell detection expected from single-plane imaging, which scales λ without altering the Poisson form. The observed distribution was consistent with the expected Poisson model under a likelihood-ratio test (X^2^(1) = 1.62, p = 0.20), with ~80% power to detect a variance-to-mean ratio of 1.38 and ~40% at 1.23 (binning above 4 CPD results in slight underestimation of variance-to-mean ratios).

Our findings showed that our pipeline enabled precise encapsulation and tuning of bacterial density within droplets. The agreement between the observed puncta distribution and the expected cell distribution within medium-density droplets suggested we could indeed resolve single cells. Taken together, our results also suggested that by starting at low cell densities, we could detect and quantify growth of fluorescently labeled *E. coli* during time-lapse imaging, making multiplexed tracking of growth within individual droplets possible.

### Simplified multiplexed growth tracking reveals sigmoidal growth profiles and heterogeneity in growth rates

We next attempted to track growth of *E. coli* within individual droplets embedded in agarose. We generated droplets using an LB-grown culture at OD of 1, expecting an average of 3.65 cells per droplet. The proportion (2.9%) of manually observed empty droplets at T = 400 minutes (11) to all manually observed droplets at T = 0 (384) approximated the expected proportion of empty droplets based on Poisson statistics (2.6%).

We tracked all detected droplets falling within a chosen X-Y axis window within the full FOV to avoid cropping, and recorded data only from those in which the tracking software successfully recorded pixel intensity at all timepoints (N = 299). Pixel intensities within droplets increased over a period of six hours, consistent with cell growth (Fig 3A, 3B). Using FIJI’s TrackMate software, we recorded the mean pixel intensity value within every detected droplet in a given FOV over six hours, as a proxy for cell growth. Mean pixel intensities for most droplets followed a common sigmoidal shape, consistent with expected growth profiles (Fig 3C). Averaging mean pixel intensity values for all droplets at 5-minute timepoints also generated a sigmoidal curve resembling OD over time plots for batch bacterial cultures moving through lag, log, and stationary phases. On average, the exponential growth phase occurred roughly between 125 and 200 minutes into the experiment, with the maximum average growth rate across all droplets (0.82 doublings/hour) occurring at T = 155 minutes (Fig 3D). Repeating the analysis in triplicate showed consistent growth curves across FOVs (S7 Fig); Peak average growth rates were 0.88 and 0.87 doublings/hour in FOVs 2 and 3 (N = 399 and 406 droplets), respectively.

**Fig 3:**
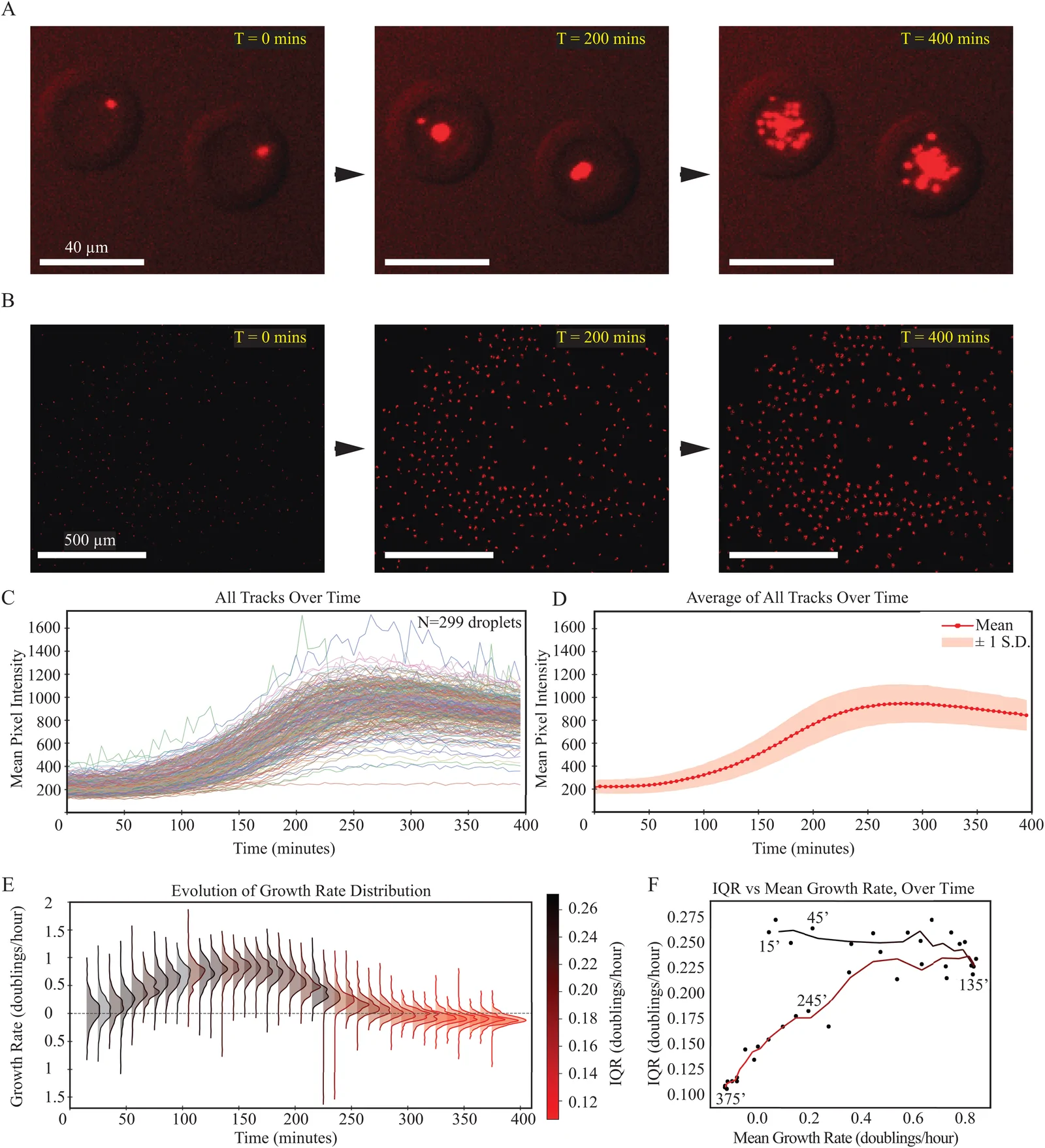
Quantitation of growth rate among single droplets reveals steady and then decreasing variation in growth rate as a function of culture phase. (A) Representative zoomed-in fluorescence images of mScarlet1-expressing *E. coli* within 2 droplets at 0, 200, and 400 minutes. Increase in pixel intensity over time is consistent with cells growing and dividing inside droplets. (B) Representative fluorescence images of mScarlet1-expressing *E. coli* within all droplets within a single FOV at 0, 200, and 400 minutes. Detection and tracking of cell growth in many agarose-embedded droplets in parallel. (C) Mean pixel intensities for 299 individual droplets over time; each droplet is shown in a different color. Tracking changes in pixel intensity reveals growth profiles consistent with expected sigmoidal growth. (D) Mean pixel intensity averaged across 299 droplets over time; red line indicates the mean and shaded area indicates ± 1 standard deviation across all growth tracks. The averaged profile resembles the expected sigmoidal curve from batch assays. (E) Growth rate PDF time-series colored by IQR (spread of middle 50% of the data), showing a progressive narrowing of the spread in the growth rate distributions. Dashed line represents a growth rate of 0 (stationary phase) (F) Scatterplot of IQR as a function of mean growth rate over time; Each point represents a single timepoint (15–375 min, 10-min intervals; N = 37) from a FOV containing 299 tracked droplets, plotted as IQR (y-axis, doublings/hour) versus mean growth rate (x-axis, doublings/hour). IQRs were computed across all droplets within the FOV at a given timepoint. Lines connect timepoints using a 3-point smoothing to illustrate temporal trajectories, with color transitioning from black (early) to red (late). Representative timepoints are denoted by time annotations in minutes (‘). Across the time course, IQR remains mostly constant until maximal average growth rate is reached and then decreases as growth begins to slow down.

Next, we wondered if our method could reveal growth rate heterogeneity. Because each droplet serves as an independent, parallel culture, differences in growth rate across droplets might reveal such heterogeneity. We calculated the growth rates (doublings per hour) within individual droplets at a given time point by averaging across 30-minute windows. We then plotted growth rate probability density functions (PDF) in 10-minute increments. We observed an initial increase in the mean growth rate over time followed by a convergence towards zero in the stationary phase (Fig 3E). We also noticed a decrease in both mean pixel intensity and growth rate, with the latter becoming negative at late timepoints.

Variation in growth rate across droplets held steady as cells started growing, then decreased as cells stopped growing. Comparing the interquartile range (IQR) between “early growth” (0–115 minutes) and “late growth” (165–275 minutes) periods with equal growth rates (0.41 doublings/hour) revealed a steady decrease in the spread of growth rates over time (early average IQR = 0.25 vs late average IQR = 0.20). Such differences were amplified when comparing individual timepoints with similar growth rates. IQR was 44% higher at the early timepoint than at the late one, at roughly equal growth rates of ~0.20 doublings per hour (IQR of 0.26 at 45 minutes vs IQR of 0.18 at 245 minutes) (Fig 3F).

To better understand the observed heterogeneity, we performed additional analysis aiming at controlling for the effect of noise, background, and differential cell-loading caused by Poisson statistics. First, we defined a “biomass index” (S0), measured as the average background-subtracted signal over the first five frames. While S0 is an indirect proxy for cell abundance within droplets, droplet-to-droplet differences greatly exceed measurement noise, making it a reliable stratifier at initial timepoints. We used S0 to divide our N = 299 sample into “low,” “mid,” and “high” density terciles at T = 0 (S5 Fig). To control for background and detection noise, for this analysis we subtracted each droplet's own background from every frame rather than from the baseline alone. As a consequence, apparent growth rates increased and the population maximum advanced to a 30-minute window spanning 110–140 minutes, which we therefore used for the tercile comparison. Comparing mean growth rates at this window revealed a decline confined to the highest tercile, with 1.30 and 1.26 doublings/hour in the low and mid terciles (Dunn's test, p = 0.19) against 0.95 doublings/hour in the high tercile (Kruskal-Wallis H = 119.6, p = 1.1 × 10^–26^, ε² = 0.40, Spearman ρ = −0.61 between initial biomass and growth rate, N = 299).

To understand whether the variation in growth rates persists in the absence of differential cell loading, we manually selected 29 droplets containing single cells from each FOV, based on observed puncta counts at T = 0. We calculated 95% confidence intervals for each droplet's growth rate within the 130–160 minute window, and observed that 8 of 29 (28%) and 6 of 29 (21%) intervals excluded the mean growth rate among single-cell droplets in FOVs 2 and 3 respectively, against the expected 5% if all droplets shared a single true growth rate (binomial p = 1 × 10^–4^ and 3 × 10^–3^) (S5 Fig). In FOV 1, 3 of 29 intervals (10%) excluded the mean, which does not differ significantly from expectation (p = 0.18). Across all three FOVs, the standard deviation of growth rate among single-cell droplets was 84%, 118% and 119% of that measured across all droplets in the same field, indicating that restricting to a fixed starting cell number removed little of the observed variation.

## Discussion

The complexity, accessibility and cost involved in trapping and tracking droplets over time poses an obstacle for the broad adoption of droplet microfluidic technologies. Overcoming these challenges often requires compromising throughput. Here, we developed a simple-to-implement pipeline for long-term imaging and tracking of double emulsion (DE) droplets. Our method addresses three common goals in droplet-based technologies: (i) maintaining droplet position within a field of view during extended imaging assays, (ii) minimizing the complexity involved in trapping DE droplets, and (iii) avoiding the development of custom tracking software, while maximizing throughput.

We prevented excessive droplet shifting by using glass-bottom dishes holding an abundance of buffer sufficient to minimize impacts of evaporation. We also took advantage of an inherent property of DE-droplets, an aqueous outer phase, to implement an optional agarose-based trapping step, further stabilizing droplet positions against accidental jostling; no detectable changes in the droplet core or shell diameter were observed throughout our experiments. We conducted all image processing and data analysis with free-to-use and open-source software [38].

To establish our method, we demonstrated that either approach can reduce droplet shifting within a given field of view (FOV) for up to 12 hours. Next, we showed that for droplets containing bacteria we could control the density of cells within droplets by adjusting the optical density (OD) of input cultures. We observed apparent Poissonian distributions of cells per droplet, suggesting our simple single-plane imaging method enables quantitation of bacteria approximating single-cell resolution. Lastly, we leveraged our method to monitor the growth of bacteria within many droplets in parallel, revealing heterogeneity in growth rates. Because cell distribution among droplets follows Poisson statistics, some of this spread could reflect differential loading at T = 0 rather than differences in growth rate between cells. Restricting the analysis to droplets containing single cells removed almost none of the observed heterogeneity in growth rates (S5 Fig), consistent with the effect of initial biomass being confined to the highest occupancy tercile. Variable cell loading therefore accounts for little of the droplet-to-droplet spread in growth rate.

Previous droplet-monitoring efforts have either neglected individual droplet identity, or have required complex secondary trap-devices, which increase complexity and cost while reducing throughput [25,39–42]; space occupied by structural traps reduces droplet density in the FOV. Our method is compatible with fully packed FOVs, maximizing throughput while eliminating the need to build specialized secondary devices. Our simple imaging set-up includes the use of widely accessible ImageJ software. While previous methods have successfully tracked single-emulsion droplets using ImageJ [2], our pipeline is the first to implement standard tracking software for DE-droplets in the absence of a dedicated trapping device. The benefits of our image analysis and tracking method also include reduced data analysis time (minutes) compared to some previously reported methods (days) [31].

Our pipeline has three inherent limitations. First, our method is not designed to track individual cells over time but instead tracks total cell growth within many individual droplets in parallel. Measurements based on pixel intensities relating to a fluorescent reporter serve as a proxy for cell density, limiting our capacity to make absolute quantitative claims. Factors such as signal linearity at high densities, maturation rates unique to any given fluorophore, and the stability of such markers set a limit on our method’s resolution. Second, the bulk arrangement of droplets within a sample hinders multiplexed treatment testing. Although built-in dividers allowed us to test four agarose concentrations in parallel within a single dish (S2 Fig), multiplexed condition testing remains low throughput. Third, the agarose-free implementation of our method is vulnerable to mass shifting induced by accidental bumping or shaking of the microscope stage or dish during imaging; future work could include a detailed analysis of the relationship between agarose concentration and droplet displacement.

We observed a modest decrease in pixel fluorescence during late timepoints; comparing our data to plate-based bulk OD measurements suggests that this phenomenon is not unique to our imaging set-up and is consistent with growth curves seen outside droplets (S6 Fig). The growth of our *E. coli* cells seems consistently slower than what is expected for wild-type strains. The slower growth is most likely due to the use of a strong constitutive promoter expressing the mScarlet1 reporter from a high-copy plasmid (S6 Fig).

Our method is theoretically limited by the number of droplets that can be packed within a given FOV (~1,500 40µm-wide droplets at 10X magnification) and the number of FOVs imaged between timepoints. Here we tracked bacterial growth within 1,104 droplets across three FOVs for up to 6.6 hours at five-minute intervals. Built-in dividers allow up to four conditions to be run within a single dish. Each experiment consumes one glass-bottom dish (Cellvis D35C4-20–0-N, ~ $2.30) and ~$0.05 of low-melt agarose (1–4 mg at 0.25–1% w/v in 400 µL of outer buffer), for a total consumable cost under $2.50 per run, or roughly $0.60 per condition when all four dish chambers are used. All image processing was performed in FIJI/TrackMate, which is free and open source. Using modified microscope stages capable of holding additional dishes, or the use of glass-bottom dishes with more dividers could help increase the number of conditions tested in parallel. Performing Z-stack imaging at high magnification may provide an opportunity to detect and track individual cells over time.

Studying individuals comprising a population is particularly exciting for phenotyping large variant libraries. Ensuring encapsulation of single entities within droplets through Poisson statistics requires working with input densities yielding mostly empty droplets. Reducing the percentage of droplets carrying two or more molecules to roughly 1% requires ~86% of all droplets to be empty. As a result, only ~14% of droplets are available for single-entity observations. This “Poissonian poverty” suggests that active methods for loading individuals into droplets could be useful.

Our work addresses a need for further scaling of droplet monitoring methods, which will enable a deeper understanding of the individual biological and molecular behaviors comprising population-level observations. Our results showcase the potential of simplified methods as drivers in the improvement and adoption of droplet microfluidic technologies for high-throughput phenotyping. Only by seeing the behavior of individuals comprising populations can we hope to find individual specimens satisfying specific needs and realize a more complete understanding of populations as a whole.

## Methods

Extended methods are available in the Supplemental Online Material associated with this manuscript (https://osf.io/3nkyw).

### Double-emulsion droplet generation

We generated double emulsion droplets in PDMS microfluidic devices using previously reported architectures [24,28]. The chip design files along with a fabrication protocol can be found in our public repository (https://osf.io/3nkyw). Our device featured four inlets: one for the outer aqueous phase (inlet #4), one for the oil phase (inlet #1), and two for the inner aqueous phase (inlets #2 and 3). For the initial agarose trapping optimization, we generated “empty” droplets carrying PBS pH 7.4 (Gibco) + Tween-20 (Fisher) with an oil shell of HFE7500 + 5% PicoSurf (Sphere Fluidics)(v/v). Using blunt-tip needles (Millipore Sigma), we connected 1 mL plastic syringes (BD) carrying the inner solutions to LDPE medical tubing (Scientific Commodities Inc.) that were directly connected to the PDMS device. Syringe pumps (Harvard Apparatus) controlled the flow of reagents into the device, with standard flow rates of 2 × 25 μL/h for two identical inner solutions of PBS (Gibco) with 1% (w/v) Tween-20 (Fisher), 125 μL/h for the fluorocarbon oil phase with 5% PicoSurf in HFE7500 (Sphere Fluidics)(v/v), and 3500–3700 μL/h for the outer sheath solution of PBS with 1% (w/v) Tween-20 and 2% (w/v) Pluronic F68 (Millipore Sigma). To encapsulate bacteria in droplets, we introduced suspensions of bacteria in LB medium through both inner inlets at equal flow rates. For the cell-density calibration experiments (Fig 2, S3 Fig), the second inner inlet instead carried sterile LB, diluting the culture 1:1 on chip. To slow bacterial growth until imaging, we stored droplets on ice or at 4 ^°^C for up to 1 hour.

### Agarose preparation and droplet trapping

After droplet generation, we fully dissolved low-temp melt agarose powder (Invitrogen) at the indicated concentrations in outer phase solution (PBS or LB with 1% (w/v) Tween-20 and 2% (w/v) Pluronic F68 (Millipore Sigma)). We fully dissolved the agarose solutions and kept at 45 ^°^C until the trapping began. We first deposit 400uL of agarose solution into a dish well, allowing it to cool down to around 37 ^°^C. Next, we used a 200uL pipette to deposit 10uL of a droplet sample at the bottom of the agarose layer, right at the glass interface. We then gently swirled the dish to ensure the droplets settled on top of the glass surface as a highly confluent monolayer before agarose solidification, which begins at around 25 ^°^C. We allowed the dish to sit at room temperature for 5 minutes before introducing it into a pre-warmed, temperature-controlled microscope at 37 ^°^C for long-term imaging.

### Strains and growth conditions

We generated the input bacterial stocks used throughout our experiments by inoculating *E. coli* carrying an mScarlet1 + Kanamycin resistance cassette from a glycerol stock into LB + Kan. The inoculate grew at 37 ^°^C, shaking at 250 RPM overnight to saturation. We placed the cultures on ice for 10 minutes before measuring their OD. Next, we placed 1 mL aliquots into 1.5 mL Eppendorf tubes, and spun down at 1000 x g in a chilled centrifuge (4 ^°^C) for 5 minutes. We discarded the supernatant, then we washed and re-suspended the pellet with a volume of fresh LB that would yield the desired dilution or concentration factor. Careful up-and-down pipetting broke apart cell clumps from the pellet and avoided clogging the microfluidic device. For the plate-based bulk OD measurements, the same sample preparation was performed, with 50uL of OD = 1 culture being placed per well in triplicate within a 96 well plate. OD was obtained at 10-minute intervals with the Spectramaxi3’s OD600 detector using continuous orbital shaking at medium intensity, maintaining a temperature of 37 ^°^C.

### Microscopy and image processing

We captured brightfield and fluorescence images on an inverted light microscope (Nikon) with an Oko temperature control chamber set to 37 ^°^C. All images were taken at 10X magnification at either 2 minute (for standard imaging set-up demonstration) or five-minute intervals (for long-term tracking). We obtained image stacks by imaging several fields of view within a given sample in five-minute intervals. We performed image processing using FIJI software. We used the background subtraction function for flat-field correction, using a 25-pixel rolling-ball. We performed droplet detection, tracking and fluorescence quantification, using the TrackMate’s LoG detector, with an estimated object diameter of 50 pixels. During detection, we set a quality score filter to 0.3, aiming to strike a balance between maximizing true droplet detection and minimizing false positives. For the FOV reported on main Fig 3, the quality score filter resulted in 365 droplets detected at T = 0. We next used positional filters to eliminate any droplets that seem too close to the edges, since this may cause unreliable signal due to cropping, keeping only droplets sitting between pixels 45.93 and 1994.51 on the X coordinate, and between pixels 39.37 and 1935.46 on the Y-axis (TrackMate is capable of sub-pixel localization). Our positional filters reduced the number of detected droplets to N = 299, all of which were detected across all 80 frames. We selected the Simple LAP tracker, setting the linking max distance to 50 pixels, the gap-closing max distance to 10 pixels, and the gap-closing max frame gap to 0 frames (S4 Fig). We used custom python scripts for all downstream data analysis and plotting.

### Statistical analysis

Per-droplet growth rates were obtained by least-squares regression of log_2_ (mean pixel intensity) against time, giving a slope in doublings per hour. For time-resolved analyses, rates were computed in 30-min windows (7 timepoints) advanced in 10-minute steps across the time course, and dispersion at each window summarized as the interquartile range across droplets (Fig 3E, 3F). For fixed-window comparisons, the analysis window was selected by sliding the same 30-min window in 5-minute steps and taking the window with the highest mean growth rate across all droplets (130–160 minutes, centered on minute 145). Initial “biomass” (S₀) was defined per droplet as the mean background-subtracted signal over frames 0–4, where background was that droplet's mean MEDIAN_INTENSITY_CH1 over the same frames, floored at 1; droplets were assigned to equal-count low, mid and high terciles of S₀. For the tercile comparison, this per-droplet background was additionally subtracted from every frame before fitting; the comparison window was again the one with the highest mean growth rate across all droplets, which under this correction fell at 110–140 minutes, centered on minute 125. The standard error of each droplet's fitted slope was derived from the residual scatter about its own regression line (5 degrees of freedom), and 95% confidence intervals calculated as slope ± t (0.975, 5) × SE, with t = 2.571. A droplet's growth rate was classed as differing from the average when this interval excluded the mean growth rate; under the null hypothesis that all droplets share a single true growth rate ~5% of intervals would do so, and observed counts were compared against this expectation by binomial test. Differences in growth rate across biomass terciles were assessed by Kruskal–Wallis test with ε^2^ as the effect size and Dunn's test with Holm correction for pairwise comparisons, and the monotonic association between S₀ and growth rate by Spearman rank correlation. Agreement between observed cells-per-droplet counts and a Poisson model was tested by likelihood-ratio test against a negative binomial alternative. All analyses were performed in Python 3 using numpy, pandas, scipy and scikit-posthocs; analysis code and source data are available at https://osf.io/3nkyw.

## Supporting information

S1 Fig Droplet trapping protocol. (A) 400 µL of molten agarose at 45 ºC are pipetted into a glass-bottom dish. (B) Droplets are injected into the bottom of the dish while agarose is still liquid (C) Dish is swirled to help droplets fall to the glass surface and rest as a confluent monolayer before agarose solidification. (D), (E), and (F) show successfully trapped droplets from different angles. Red circles mark the droplet monolayer.
(EPS)

S2 Fig Microscopy shows droplets are compatible with agarose embedding for up to 12 hours. (A) Droplets fixed in PBS-containing agarose at concentrations ranging from 0 to 1% W/V. All conditions were tested in a glass-bottom dish using 400uL of outer buffer. Each row represents a different agarose concentration. Each column represents a unique timepoint.
(EPS)

S3 Fig Microscopy of empty and high-density droplets tracks expected Poisson distributions. (A) Merged brightfield and fluorescence images of droplets at different cell-density regimes (left to right: OD_600_ of 0, 0.84, and 9.4). (B) Expected Poisson distribution of cell occupancy within droplets for each cell density regime.
(EPS)

S4 Fig FIJI data analysis pipeline. (A) Steps for droplet detection and tracking in FIJI within the graphic user interface (GUI). (B) Images depicting progression of hyperstack before and during analysis using TrackMate’s GUI. Images i and ii are obtained before executing TrackMate, using FIJI’s background subtraction tool.
(EPS)

S5 Fig Growth rate heterogeneity persists among droplets containing single cells. (A) Biomass Index captures spread of baseline signal across droplets, which vastly outsizes noise within droplets. (B) Biomass terciles show significant differences in growth rate, with high density droplets exhibiting lower average growth rates. (C) Mean pixel intensity over time for droplets containing single cells, showing sigmoidal growth profiles comparable to those of the full droplet population. (D) Per-droplet 95% confidence intervals on growth rate for single-cell droplets; more intervals exclude the single-cell mean than the 5% expected if all droplets shared a single true growth rate. (E) Distribution of growth rates among single-cell droplets compared with all droplets in the same field; restricting to a fixed starting cell number does not reduce the spread.
(EPS)

S6 Fig Plate-based bulk OD measurements recapitulate in-droplet growth curves. (A) Normalized growth curves for mScarlet1-expressing *E.coli* cells show consistency regardless of growth and tracking method, with similar maximum growth rates being reached around the same time-point (155 minutes). Both cultures had a starting OD of 1. The red curve is the mean pixel intensity across the 299 tracked droplets, min-max normalized to 0–1, shaded by the standard deviation across droplets on the same scale. The black curve is the blank-subtracted absorbance of the three bulk wells, normalized the same way and shaded by the standard deviation across wells. Each series carries its own time column, since the microscope and plate reader sampled on grids offset by 5 min. Growth rates were fitted to the unnormalized measurements, not the normalized curves.
(EPS)

S7 Fig Growth tracking is consistent across fields of view. (A–C) Mean pixel intensity over time for every tracked droplet in FOV 1–3 (n = 299, 399 and 406 droplets); each droplet is shown in a different color. (D–F) Mean pixel intensity averaged across all droplets in each FOV, min–max normalized to 0–1; red line indicates the mean and shaded area indicates ± 1 standard deviation across tracks. Dashed lines mark the maximum average growth rate in each FOV (0.82, 0.88 and 0.87 doublings/hour, at 155, 135 and 145 minutes respectively).
(EPS)

## Abbreviations

CPD: cells per droplet
DE: double emulsion
FOV: field of view
IQR: interquartile range
LDPE: low density polyethylene
OD: optical density
PBS: phosphate buffered saline
PDF: probability density function
PDMS: polydimethylsiloxane
SE: single emulsion
